# Development and Adoption of Genetically Engineered Plants for Virus Resistance: Advances, Opportunities and Challenges

**DOI:** 10.3390/plants10112339

**Published:** 2021-10-29

**Authors:** Prakash M. Niraula, Vincent N. Fondong

**Affiliations:** Department of Biological Sciences, Delaware State University, Dover, DE 19901, USA; pniraula@desu.edu

**Keywords:** genetically engineered (GE), genetically engineered organism (GMO), regulation of GMOs, virus-resistant transgenic crops

## Abstract

Plant viruses cause yield losses to crops of agronomic and economic significance and are a challenge to the achievement of global food security. Although conventional plant breeding has played an important role in managing plant viral diseases, it will unlikely meet the challenges posed by the frequent emergence of novel and more virulent viral species or viral strains. Hence there is an urgent need to seek alternative strategies of virus control that can be more readily deployed to contain viral diseases. The discovery in the late 1980s that viral genes can be introduced into plants to engineer resistance to the cognate virus provided a new avenue for virus disease control. Subsequent advances in genomics and biotechnology have led to the refinement and expansion of genetic engineering (GE) strategies in crop improvement. Importantly, many of the drawbacks of conventional breeding, such as long lead times, inability or difficulty to cross fertilize, loss of desirable plant traits, are overcome by GE. Unfortunately, public skepticism towards genetically modified (GM) crops and other factors have dampened the early promise of GE efforts. These concerns are principally about the possible negative effects of transgenes to humans and animals, as well as to the environment. However, with regards to engineering for virus resistance, these risks are overstated given that most virus resistance engineering strategies involve transfer of viral genes or genomic segments to plants. These viral genomes are found in infected plant cells and have not been associated with any adverse effects in humans or animals. Thus, integrating antiviral genes of virus origin into plant genomes is hardly unnatural as suggested by GM crop skeptics. Moreover, advances in deep sequencing have resulted in the sequencing of large numbers of plant genomes and the revelation of widespread endogenization of viral genomes into plant genomes. This has raised the possibility that viral genome endogenization is part of an antiviral defense mechanism deployed by the plant during its evolutionary past. Thus, GM crops engineered for viral resistance would likely be acceptable to the public if regulatory policies were product-based (the North America regulatory model), as opposed to process-based. This review discusses some of the benefits to be gained from adopting GE for virus resistance, as well as the challenges that must be overcome to leverage this technology. Furthermore, regulatory policies impacting virus-resistant GM crops and some success cases of virus-resistant GM crops approved so far for cultivation are discussed.

## 1. Introduction

To ensure food security as the global population increases in a changing climate, there is a need to improve crop production, which continues to face multiple constraints. Viral diseases constitute one of the major threats to crop production worldwide [1], and the inability to control these viruses using agrochemicals is a serious limitation. Although conventional breeding has, and continues to play an essential role in crop improvement, it usually entails growing and examining large populations of crop plants over multiple generations. This is an expensive, lengthy, and a labor-intensive process. Moreover, occasionally, no natural sources of virus resistance exist, and even when they do, introgression of such resistance genes into some crops may be prohibitively difficult. Additionally, selecting for genetic resistance against multiple viral diseases simultaneously while maintaining the strong agronomic traits of elite cultivars may be intractable [2]. Thus, other options must be sought to contain current and future viral epidemics. One of the strategies having considerable traction is genetic engineering (GE), which overcomes most of the limitations of conventional breeding. GE is the direct alteration of an organism’s genetic material using recombinant DNA techniques and biotechnological procedures. GE methods continue to be improved due to advances in genomics and biotechnology that for some crops, allow a relatively easy transfer of DNA between organisms belonging to different species, thus overcoming the prezygotic barrier. GE has therefore provided an unprecedented opportunity to counter plant diseases and pests that are estimated to cause close to 30% losses [2,3].

The use of recombinant DNA techniques to genetically engineer plants was first reported in 1983 [4,5,6]. Tomato became the first genetically modified (GM) crop to be deregulated for commercialization by the U.S. Food and Drug Administration (FDA) in 1992 [7]. This was followed by the release of other GM crops notably soybean, maize, and cotton [8]. Since then, cultivation of GM crops has increased every year, including 12 years of double-digit growth between 1996 and 2014 [9]. Today, GM crop cultivation covers a total area of 2.5 billion hectares in 26 countries, 21 of which are developing countries [10,11]. In terms of adoption, as of the year 2019, a total of 71 countries have adopted GM crops. Such an expansion is intended to help meet the high demand for the production and distribution of nutritious food.

Engineering plants for virus resistance has grown considerably since evidence emerged in the 1980s showing that viral genes can be used to engineer plants for antiviral resistance [12], as elucidated for tobacco mosaic virus (TMV) [13]. This was followed by the development of virus resistant tobacco expressing the coat protein (CP) gene of several plant viruses [14,15]. The finding in the late 1990s that this mode of resistance was due to the newly discovered RNA silencing further expanded the use of GE to control plant viruses. The first commercially produced virus resistant GM crop was squash, which exhibited resistance to watermelon mosaic virus (WMV) and zucchini yellow mosaic virus (ZYMV) [16,17]. This was followed by transgenic papaya with resistance to papaya ringspot virus (PRSV) [18]. Today, about seven GE crops with enhanced virus resistance have been approved for commercial production in the United States of America (USA) and China, and greenhouse and field trials of transgenic plants with resistance to viruses are underway in many other countries, especially in South America and Africa [19,20].

## 2. Functional Genomics and Genome Editing in Engineering Crops for Virus Resistance

Many genetic modification strategies have been successfully used to control plant viruses in laboratories, greenhouses, and to a limited extent, in field trials. Since its elucidation in 1990, many RNA and DNA viruses have been successfully controlled using RNA silencing approaches, which suppress gene expression in a sequence-specific manner. Many RNA silencing precursors, including: sense/antisense, small interfering RNA (siRNA), microRNA (miRNA), and hairpin RNA (hpRNA) have been harnessed to generate virus resistant transgenic crops [21,22]. Some of the viral genes used to produce hpRNA constructs for processing into the silencing complex include genes coding for the CP, replication associated protein (Rep), RNA-dependent RNA polymerase (RdRP), movement protein (MP), and proteases. The RNA transcripts produced from these gene constructs are processed into small interfering RNAs (siRNAs) that are key antiviral molecules [23,24]. More recently, the shortcomings of the hpRNA strategy have at least partially been addressed using artificial microRNAs (amiRNAs) and trans-acting siRNAs (tasiRNA) based strategies that are high-throughput approaches, which are amenable to multiplexing and have been used to simultaneously control diverse RNA and DNA viruses of different families [25,26,27,28,29,30].

Non-viral antiviral genes, including especially R genes and ribosome-interacting proteins, as well as protease inhibitors have also been incorporated into crop genomes to generate resistance against viruses. Mechanistically, most of these non-viral genes silence host genes that are involved in virus replication [28,29,30,31]. Other gene silencing approaches have developed virus resistance in insect vectors in order to limit virus transmission [32]. The tasiRNA based microRNA-induced gene silencing (MIGS) strategy is another recent approach used to control multiple viruses or pests and pathogens [33].

Application of GE in virus management has also been facilitated by the advancements and breakthroughs in genome editing. This has included the use of site-specific nucleases to target the viral genome or host plant factors that are involved in the viral infection cycle. Four major classes of site specific nucleases (SSNs) that have successfully been employed to engineer virus resistance are meganucleases, zinc finger nucleases (ZFNs), transcription activator-like effector nucleases (TALENs), and more recently, the clustered regularly interspaced palindromic repeats/ CRISPR-associated 9 (CRISPR/Cas9) technology [34,35]. In early genome editing efforts, ZFNs and TALENs showed some promise in targeting begomoviruses [36,37,38]. However, the limitation with these technologies is tailoring the DNA binding protein to target the viral sequence. The emergence of CRISPR/Cas system has considerably reduced this limitation and has been the prime gene editing tool on many ongoing research efforts [34,35,39,40,41].

In plants, the CRISPR/Cas system was first used successfully to generate geminivirus resistance in both model and crop plants, including beet curly top virus (BCTV) and merremia mosaic virus (MeMV). This system was shown to simultaneously target multiple begomoviruses, including wheat dwarf virus (WDV), tomato yellow leaf curl virus (TYLCV), tobacco curly shoot virus (TbCSV), bean yellow dwarf virus (BeYDV), where the guide RNA (gRNA) was designed from the intergenic region (IR), or CP and Rep genes, [40,41,42]. CRISPR/Cas has also been used to modify plant translational regulatory genes; namely, eukaryotic initiation factor 4E (eIF4E) and its isoform (eIFiso4E) that are directly involved in virus infection. This modification resulted in resistance to both DNA and RNA viruses [43,44,45].

## 3. Benefits of Genetic Engineering in Plant Virus Control

As discussed above, GE has provided new opportunities in plant virus control efforts by addressing most of the shortcomings of conventional breeding and agronomic practices. As presented here, some of the advantages of GE for virus resistance include short lead time, relatively low production cost, high efficacy, ease of interspecies gene transfers, no major effect on the genetic background of the cultivar and can be used in vegetatively propagated crops.

### 3.1. Short Lead Time

One of the major limitations of plant breeding, which so far is the most effective plant virus management approach, is the length of time required to produce a resistant plant. This contrasts with the relatively short plant transformation lead time, even for very recalcitrant crops such as cassava. Today, the transformation process involves designing the gene construct and its transfer in the regenerative tissue of the target plant. The transgene can be synthesized and used for transformation within a week or two after being designed. Moreover, there are institutional core facilities that are specialized in transforming specific crops, which depending on the crop, can take weeks to several months in generating the transformed lines. This contrasts with conventional breeding, which can take up to 20 generations of selection and may be complicated if there is a tight genetic linkage between the desired resistance gene(s) and the genes that confer undesirable traits [46,47].

### 3.2. Comparatively Low Production Cost

The relatively low cost of production of virus resistant transgenic plants is partly due to the short turnaround time of the plant transformation process, as well as low manpower input. Furthermore, as discussed above, the advent of core facilities specialized in plant transformation has reduced duplication of purchases of expensive instruments used in plant transformation (plant growth facilities, electroporators, sonicators and gene delivery systems). These core facilities also have specialized technical knowledge for specific crops; this significantly reduces the time and effort required to construct transgenes and transform plants with different levels of amenability to transformation in modest labs. In the USA, the cost of transformation and transgenic development in potato and tomato, for example, are currently ~$1700 per construct and ~$1500, respectively.

The need for backcrossing to introduce the transgene into the most desirable plant genotypes, which are typically not amenable to transformation, has been suggested [47]. However, with advances in transformation technologies, even recalcitrant genotypes such as maize inbred lines [48], sorghum [49], and cassava [50] are transformed with relative ease.

### 3.3. Efficacy and Durability of GE-Mediated Virus Resistance

Advances in genomics have provided new pathways that can be harnessed to produce transgenes that specifically target viruses with RNA or DNA genomes. The early generation of transgenic constructs were produced using whole genes or large segments of the viral genome [13]. These transgenes were typically designed to overexpress coat proteins, mutants of movement proteins/replication associated proteins, or to produce antisense RNA, or dsRNA by either bidirectional transcription or by transcription of an inverted repeat or “hairpin”. The dsRNA template is then processed into siRNAs, which direct targeting and silencing of the cognate viral transcript. However, frequently, these constructs failed to fold correctly into dsRNA for siRNA processing. Even when there is correct folding, the siRNA produced might not efficiently target the virus or it might target an endogenous gene, thus becoming toxic to the plant.

**Figure 1:**
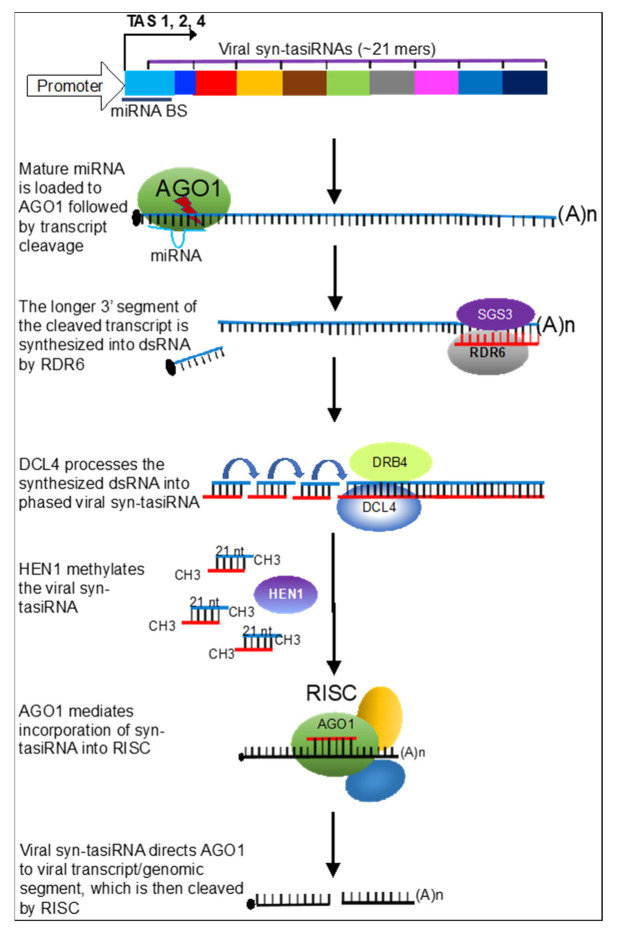
Modification of three *TAS* genes (TAS1, TAS2, and TAS4) [55] for viral resistance. Synthetic trans-acting siRNAs (syn-tasiRNAs) designed from conserved regions of one or more viral genomes are used to replace endogenous tasiRNAs and then placed downstream of the miRNA-binding site (miRNA BS) of the *TAS* gene [25,26,27]. After miRNA-mediated cleavage of the transgene, the cleaved transcript is synthesized into a dsRNA template, which is processed into syn-tasiRNA by Dicer-like 4 (DCL4). Mature syn-tasiRNAs are then loaded to the RNA-induced silencing complex (RISC) to direct the targeting of transcripts from infecting cognate virus(es). Some of the proteins involved in this pathway are represented with colored ovals.

These drawbacks have been overcome with the recent elucidation of tasiRNAs, and to an extent, artificial microRNAs (amiRNAs), both of which can be multiplexed to target multiple viruses from the same construct [27,28,51]. In either of these approaches, ~21-mer directs the silencing complex to the viral genome or transcript. Many online tools are now freely available to identify ~21-mers that would target different viruses or virus strains. As shown in Figure 1, ~21-mers synthetic tasiRNAs (syn-tasiRNAs) designed from the viral sequences can be stacked together in the same construct, thereby ensuring durability of the resistance, as it is unlikely that the multiple targeted regions of the virus(es) would simultaneously undergo mutations. The added advantage is that the ~21-mers are likely too small to recombine with infecting viruses to cause more virulent strains. Thus, the environmental risk is significantly reduced.

The advent of the CRISPR/Cas system [52] has also added to the virus-resistance toolbox. Here, the 20-mer RNA protospacer, which can be multiplexed [53] to target multiple viruses, guides Cas nuclease to target the viral genome or the host susceptibility genes.

The obvious limitation with transgenes containing ~21-mers is the apparent ease with which the target virus can mutate leading to a breakdown of the resistance. This can be mitigated by designing 21-mers from conserved regions and/or invariable motifs of viral genes.

### 3.4. Transfer of Virus Resistance Genes Is Not Limited to Closely Related Species

In conventional plant breeding, transfer of genes from distantly related species may be quite challenging because of sexual incompatibility. Given that virus resistance genes are frequently found in wild species that may not be sexually compatible with cultivated species, this becomes a serious drawback. This is the case with papaya ringspot virus (PRSV) where several wild *Vasconcellea* species (family: Caricaceae) are resistant to PRSV, but are not sexually compatible with *C. papaya* [54]. Because this incompatibility does not exist in crop genetic engineering, whole genes or small segments of genes can be transferred between incompatible species, thereby overcoming one of the insurmountable limitations in crop improvement. As discussed in Section 6 below, the PRSV problem was eventually solved by the introduction of GM papaya in Hawaii in the USA [18,54].

### 3.5. No Major Effect on the Genetic Makeup of Cultivar

Although both conventional breeding and GE involve changes in the genetic makeup of an organism with respect to DNA sequences and the order of genes, the level of genetic changes brought about by GE is much less and is well defined. This contrasts with conventional breeding where thousands of uncharacterized genes are transferred during genetic crosses. Furthermore, in GE, the gene to be introduced is selected based on the function against the target pathogen and the type of resistance mechanism to be used. Moreover, with GE, the end products, such as proteins, metabolites, as well as the phenotype are well characterized prior to release. Because in conventional breeding genomes of both parents are mixed together and randomly re-assorted into the genome of the offspring, undesirable genes are transferred along with target genes while some desirable genes are lost in the offspring.

### 3.6. Clonally Propagated Crops

To be improved using conventional breeding, the plant species must be amenable to cross-fertilization with the species containing the gene(s) for virus resistance. One of the main challenges of breeding for crop improvement is the inability or difficulty to cross fertilize. This is especially true for cultivated polyploids, for instance potato and cassava that are propagated using the vegetative mode of reproduction, as allopolyploidy combined with heterozygosity makes breeding challenging [56,57,58]. This is because during crossing between two heterozygous parents, multiple alleles segregate at both loci, and backcrossing destroys the unique gene combination within a preferred cultivar [57]. In this case, there is a possible consequence that the seeds produced are not viable. This makes GE a more effective approach to improve polyploid crops. Interestingly, vegetative propagation reduces the risk of the transgene escaping to the environment through pollen grains and thus an added advantage for GE.

From the epidemiological perspective, managing viral diseases of clonally propagated crops is more challenging due to the buildup over time of viral diseases in vegetative propagules. This partly explains why two of the most damaging plant virus diseases, caused by cassava mosaic viruses and potato virus Y, are found in clonally propagated cassava and potato. This adds to the need of adopting GE in the management of viruses of clonally propagated crops.

## 4. Potential Risks Associated with GM Crops

In spite of the benefits offered by GE for virus resistance, there are potential risks if safeguards are not followed prior to deploying this technology. These potential risks include horizontal gene transfer, genetic erosion, recombination of transgene with infecting viruses, and deleterious effect of the transgene to humans and/or animals. As addressed below, it has been scientifically documented so far that GE crops, including virus-resistant ones, do not pose undue, new, or additional risks to humans, animals, or the environment relative to crops produced from conventional breeding. Moreover, these concerns are based unfortunately on the assumption that transgenes are unnatural and inherently harmful by their very nature. Here, we discuss these risks and the way forward.

### 4.1. Horizontal Gene Transfer and Dissemination in the Environment

Concerns have been expressed about the potential of transgenes and/or selectable markers to transfer to other crops or to wild species through cross-pollination. The selectable marker gene can also be transferred to bacteria, especially in the root system, where a high rate of gene transfer with plants is known to occur [58]. Such transfers, it is suggested, may result in an ecological imbalance. This is supported by recent reports of numerous horizontal gene transfer events in eukaryotic genomes, which have been shown to be a major force propelling genomic variation [59,60,61]. However, because horizontal gene transfer occurs in nature, the debate should focus on how harmful the transgene is to the environment if it occurs. For example, engineering for virus resistance most of the time uses viral genes or genomic segments, which naturally occur in infected plants, albeit not integrated in the genome. It is highly unlikely therefore that such transgenes will cause an imbalance to the environment, at least not more than the imbalance resulting from virus devastation of cultivated crops.

As for *Agrobacterium* selectable markers, which are often antibiotic resistance genes that are included in virus-resistance transgenes, it is possible that the selectable marker can be transferred to microorganisms, thereby increasing the development of microorganisms with antibiotic resistance in the environment. However, there appears to be little indication that there is documented evidence of such transfers. In fact, in a three-year analysis of kanamycin and neomycin resistant bacterial populations collected from GM maize and non-GM crop fields, no differences in bacterial genotypes were observed. Interestingly, bacterial populations from the GM maize field tested negative for the presence of *neomycin phosphotransferase II* (*nptII)* marker gene [62]. Thus, horizontal gene transfer remains a low risk in GM crops compared with the losses caused by viral diseases.

### 4.2. Genetic Erosion

One of the concerns about large scale cultivation of transgenic crops is the possibility of a progressive depletion and loss of biodiversity as landraces are replaced by more potent GM crops. This is obviously an important concern and GE crop improvement programs must take it into consideration. Regarding engineering for virus resistance, a case can be made that introduction of virus resistance genes may preserve biodiversity by introducing resistance to highly susceptible cultivars, thereby rescuing them from virulent viruses. A classic example is the high-quality tomato variety named “San Marzano”, which is popular with the canning industry but is highly susceptible to viruses, especially cucumber mosaic virus (CMV). The absence of natural resistance to CMV almost resulted in the abandonment of this variety in the Mediterranean, especially in southern Italy [63]. Extinction of “San Marzano” was averted with the introduction of “San Marzano” tomato lines transformed with a chimeric gene expressing a benign satellite RNA of CMV [63,64]. It is unlikely that the presence of the CMV satellite in tomato will result in loss of genetic diversity in the tomato crop in the Mediterranean, in fact, the reverse may be true. Thus, it is critically important that the technology dictates decision making, not the process.

### 4.3. Recombination of Transgene with Infecting Viruses

The possibility of recombination occurring between the transgene and infecting viral genomes, giving rise to new and more devastating strains, is another risk that has been expressed regarding virus resistant transgenics. Recombination and reassortment (for segmented genome viruses) between viruses in mixed infected plants are frequent occurrences and drive virus evolution [65], as has been well elucidated for example in geminiviruses [66,67] and tospoviruses [68,69]. In each of these examples, genome recombination and reassortment of genomic segments results in the emergence of new virus variants and species. In contrast, although a spontaneous generation of hybrid virus/host DNA molecules was observed between beet curly top Iran virus and red beet [70], virus-mediated acquisition of host genetic material appears to be a rare event. Thus, it is unlikely that the transgene will specifically be transferred to an infecting virus. To the contrary, as discussed above, there is widespread endogenization of viral genomes by plants [71,72,73].

Curiously, integration of viral genomes into plant genomes is not limited to DNA viruses and retroviruses. In fact, non-retroviral integrated RNA virus sequences (NIRVs) are widespread in plant genomes [71]. While the mechanism by which NIRV RNA is converted to DNA prior to integration into the plant genome remains unknown, it should be noted that this phenomenon is also a frequent event in human and animal systems [74,75,76,77,78]. Unlike retroviruses, NIRVs do not encode a reverse transcriptase, thus, it has been suggested that NIRV integration into host genomes is through the co-option of a host reverse transcriptase [79]. Strikingly, integration of CMV RNA-1 into the soybean genome leads to production of sRNAs, which can potentially silence CMV through RNAi [80]. This discovery further stresses the fact that GE is a natural phenomenon and engineering for virus resistance is not as artificial as claimed by GM crop critics. Indeed, endogenization of viral sequences in plant genomes raises the possibility that this is an antiviral defense and would be analogous to bacterial antiphage defense using CRISPR.

### 4.4. Toxicity of Transgene

The possible adverse effects of transgenes to humans and animals, as well as to the plants have been expressed. However, an investigation of GM maize in rats either showed no negative effect [81] or the toxicity observed was due to herbicides that were used to control weed in the experiments, and not to the transgene [81,82]. With regards to GE for virus resistance, most viral resistance transgenes are designed from viral genes or segments, which are also present in foods from infected non-GM crops. In fact, a case can be made that GM crops with virus resistance contain less virus than susceptible non-transgenic crops. Additionally, new genomics and genetic engineering capabilities have enabled fine-tuning of GE such that transgenes are increasingly small and specifically targeted as found in tasiRNA and amiRNA transgenes. The bacterial marker gene *nptll*, which is routinely included in transgenes, is unlikely to cause a negative effect to humans and animals as reported in two reviews [83,84].

Toxicity of transgenes to the transgenic plant is increasingly unlikely since constructs are now routinely screened for the ability to target host genes, thanks to the availability of plant genome sequencing data. Furthermore, transgenic plants are rigorously screened for any morphological abnormalities. Taken together, the risks of transgene causing negative effects to humans and animals are overstated, especially for GM crops containing transgenes of viral origin.

## 5. Regulation of Genetically Modified Crops

Fundamentally, there are two GMO regulatory models: preventive (USA/Canada model) and precautionary (European Union model). Most other countries employ a combination of the two approaches, depending on the product being regulated and the socio-political pressures on the regulators by policy makers. In the precautionary approach the priority is to anticipate and guard against environmental contamination [85]. This approach, which is process-based, imposes strict regulations on researchers and producers rather than on the product. By contrast, the preventive approach is product-based and concentrates on identifying the damage and risks associated with the specific product, irrespective of the method used to develop it. Thus, new products and processes are screened to ensure that they do not give rise to any similar hazards that have been scientifically proven to adversely impact humans, animals, or the environment. In the preventive approach, decisions about the need for regulation and the level of regulation required are taken in relation to the relevant benefits and costs and this applies to both GM crops and conventional breeding products. These two approaches are at the basis of differences in attitudes and adoption of GM crops between the USA and Canada, and the European Union (EU).

**Figure 2:**
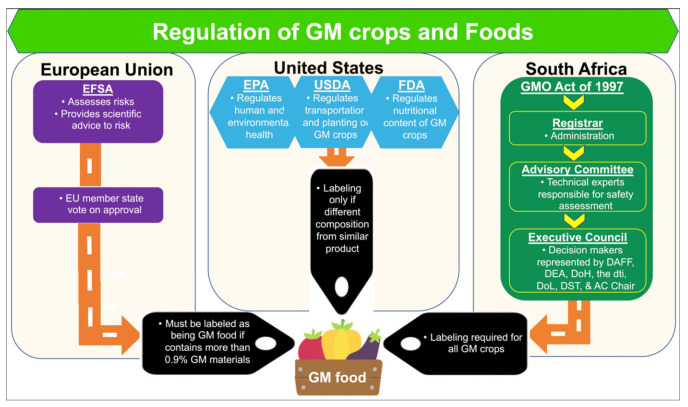
Regulatory bodies for approval of GM crops and foods in the European Union (EU), United States of America (USA), and South Africa. In the European Union and South Africa, one agency approves, while in the USA, three agencies evaluate the potential risks of GM foods. (Abbreviations: AC: Advisory Committee; DAFF: Department of Agriculture, Fisheries and Forestry; DEA: Department of Environment Affairs; DoH: Department of Health; DoL: Department of Labour; DST: Department of Science and Technology; DTI: Department of Trade and Industry; EFSA: European Food Safety Authority; EPA: Environmental Protection Agency; FDA: Food and Drug Administration; USDA: U.S. Department of Agriculture.

In the USA, three federal agencies enact regulations on both conventional and GM crops [86] (Figure 2). The Food and Drug Administration (FDA) evaluates the safety of substances added to foods, such as artificial sweeteners and genetically engineered proteins. The USDA Animal and Plant Health Inspection Service (APHIS) sets regulations to make sure that GMO plants are not harmful to other plants. USDA’s Biotechnology Regulatory Services (BRS) implements these regulations. The Environmental Protection Agency (EPA) determines the risks of pesticides and chemicals to human and environmental health, whether the substances are applied traditionally as a spray or expressed by the GM crop.

GM crop regulation in Canada is product-based as in the USA, thus, GMO crops undergo the same review process as other new agricultural products [87]. Correspondingly, several agencies are involved in the Canadian regulation process, the main regulators being Canadian Food Inspection Agency (CFIA), Health Canada, and Environment Canada, like in the USA.

In the EU, The European Food Safety Authority (EFSA) assesses and provides scientific advice to risk managers on any possible risks that the deployment (e.g., consumption or cultivation) of GM crops may pose to humans, animals, and the environment [88] (Figure 2). EFSA’s scientific advice on the risk assessment of GM crops is given through its scientific Panel on GMOs consisting of scientific experts. EFSA focuses on specific risk assessment areas addressing: (a) the molecular characterization of GM crop, (b) the safety assessment of GM crop, and (c) the environmental risk assessment of GM crop.

As for South Africa, GM crops are regulated under the GMO Act, which oversees research and development, import/export, production, consumption and other uses of GMOs and their products (Figure 2). The GMO Act establishes minimum standards to ensure the food/feed and environmental safety and socio-economic sustainability of all activities involving GMOs [89].

## 6. Some Success Cases of Virus-Resistant GM Crops So Far Approved for Cultivation

As shown in Table 1, seven GM crops with resistance to plant viruses have so far been approved for cultivation [11,90]. Five of these crops including papaya, potato, cassava, bean, and squash, which were engineered for resistance to economically important viruses, are discussed here.

### 6.1. Papaya (Resistance to PRSV Resistant)

Papaya ringspot virus (PRSV)-resistant papaya (*Carica papaya*) was one of the first virus-resistant transgenic plants to be approved in response to the PRSV epidemic in Hawaii (USA) in 1990s. This is because of the inability to develop resistance through conventional breeding to contain the epidemic. Transgenic papaya lines developed under the trade names “Rainbow” and “SunUp” and expressing the coat protein gene of PRSV were first introduced in Hawaii in 1998 [18,103,104]. Since then, these transgenic lines have exhibited broad-spectrum resistance to PRSV strains in Hawaii, but were not as successful elsewhere [90]. Additionally, because of the success of “Rainbow” and “SunUp” in Hawaii, other transgenes were developed by researchers in other countries, including a transgenic line named “Huanong No. 1” harboring the PRSV defective replicase gene, which was deregulated for commercial production in China [105]. Today, there are four transgenic papaya lines that have been approved for commercial production, three of which are grown in the USA, namely “55-1” (“Rainbow” and “SunUp”), “63-1”, “X17-2” and “Huanong No.1” in China [90]. With advances in genomics and biotechnological techniques, improved transgene constructs are being developed and tested against local PRSV strains in other countries.

### 6.2. Potato (Resistance to PVY and PLRV)

Potato leaf roll virus (PLRV) and potato virus Y (PVY) are arguably the two most important viruses of potato (*Solanum tuberosum* L.) worldwide [106,107]. Although there is natural resistance to these viruses, introgression of resistance genes to potato cultivars is demanding and time-consuming due to the highly heterozygous, outcrossing and polyploid nature of potato. Because of the increasing epidemics of PVY and PLRV in the USA between the late 1980s and early 1990s, the GE approach was deployed by transforming the commercial cultivar “Russet Burbank” with the CP gene of PVY [108,109]. The transgenic line exhibiting resistance to PVY was approved in 1998, and are marketed and cultivated in the USA and Canada under the trade name “Hi-Lite NewLeaf^TMY^” and “NewLeaf™ Y”, respectively [90,110]. This effort was followed by the transformation of the same cultivar with the two open reading frames (ORFs) of PLRV RdRp, ORF1 and ORF2, respectively, to produce two PLRV-resistant lines approved for marketing in the USA and Canada under the name “NewLeaf™ Plus” [111,112]. Overall, 14 transgenic potato lines have been approved for cultivation in North America [90].

### 6.3. Bean (Resistance to BGMV)

Bean golden mosaic virus (BGMV) and related viruses are considered the most destructive viruses of bean (*Phaseolus vulgaris* L.) production worldwide [113,114], but especially in Latin America [115]. BGMV is transmitted by the whitefly (*Bemisia tabaci*) and it causes characteristic symptoms of yellow-green mosaic leaves, distorted pods and stunted growth [116]. Bean was transformed with BGMV AC1 gene [101] from which line “EMBRAPA 5.1” that exhibited a high resistance was approved for commercial cultivation in Brazil in 2011 [117]. “EMBRAPA 5.1” has since been used as a source of BGMV resistance in crosses with Brazilian commercial bean varieties, and this effort produced a BGMV resistant cultivar approved and cultivated under the name “BRSFC401 RMD” [118].

### 6.4. Squash (Resistance to ZYMV, WMV and CMV)

Squash (*Cucurbita pepo*) was one of the first crops that was genetically engineered in the early 1990s in an effort to contain epidemics of zucchini yellow mosaic virus (ZYMV), watermelon mosaic virus (WMV), and cucumber mosaic virus (CMV) [119]. Like other early efforts in the use of GE to generate resistance to viruses, the CP genes of ZYMV and WMV were used to transform squash. A transgenic line with a strong resistance to ZYMV and WMV, designated “ZW20”, was approved for cultivation in the USA in 1994 [16]. Subsequently, the CP genes of all three viruses were used in the transformation and line “CZW-3”, which exhibited resistance to all three viruses, was approved for cultivation in 1996 [90]. These transgenic lines represented about 12% of all USA squash production and it was estimated that growers benefitted about $24 million from the technology in 2006 [120].

### 6.5. Cassava (Resistance to CBSD)

**Figure 3:**
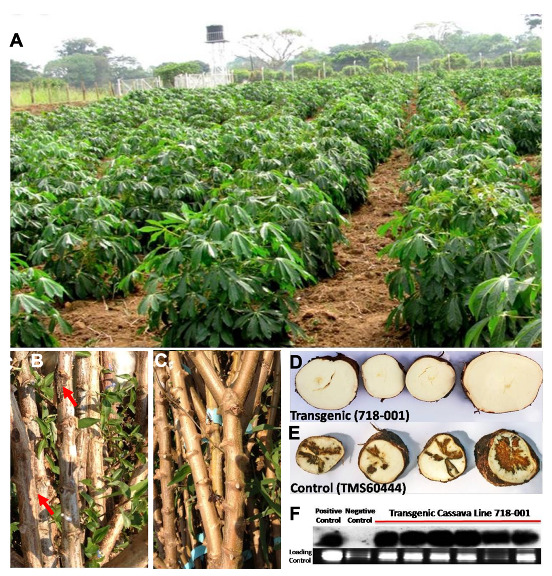
(**A**) Field resistance to CBSD in RNAi-based transgenic cassava lines in the confined field trials conducted in Namulonge research station in Uganda [125,126]; (**B**) Dark-brown necrotic lesions (arrow) seen on stems of CBSD-infected plants, but not on (**C**) in transgenic lines that exhibit resistance to CBSD; (**D**) Tuberous roots of transgenic cassava lines show no hard brown corky rot found in (**E**) non-transgenic susceptible plants; (**F**) siRNA accumulation in transgenic cassava for the full-length (FL)-ΔCP (pILTAB718) by northern blotting [126]. The negative control is RNA from a healthy non-transgenic (TMS60444) plant and the positive control is the original transgenic plant (718-001) used for propagation in the field trials (Modified from [122]).

Cassava is a staple food crop that is resilient to climate change and cultivated in several tropical regions of Africa, Latin America, and South Asia. However, it is affected by at least 20 different viral diseases, of which cassava mosaic disease (CMD) and cassava brown streak disease (CBSD) are the most damaging [121,122,123]. Most recently, in response to the devastation on cassava of CBSD in East Africa [121], a CBSD resistant RNAi-based GM cassava event 4046, developed by the Donald Danforth Plant Science Center (St. Louis, MI, USA) and other collaborators from African countries, was approved for environmental release by the Kenya National Biosafety Authority (NBA) Board [124]. The contained field evaluation and characterization of this CBSD-resistant line in East Africa are shown in Figure 3.

## 7. Conclusions

The need to achieve food sustainability and food security in an ever-increasing world population in a changing climate is central to current crop improvement efforts. Viral diseases constitute a major constraint to crop production across all geographic regions. Although conventional breeding has been key to the management of numerous viral diseases, it has its limits. Some of the disadvantages of antiviral plant breeding include absence of natural resistance, complexity, and difficulties in introgressing available resistance genes to cultivars by crossing, amount of time required to produce a cultivar, and the cost of sustaining an effective breeding program. Many of these drawbacks are overcome by the use of GE. However, the overarching challenge regarding GM crops is acceptance of the technology by the public. Yet, there has been no evidence of adverse consequences GM crops and products to human health from its consumption, as confirmed by credible international organizations [127,128].

Unfortunately, policies regulating GM crop cultivation vary considerably from country to country. The USA approach that is product-based appears to be more forward thinking, as it assesses the risks of the product and not the process. Thus, GM foods are considered not to be fundamentally different from other foods in terms of overall composition, and therefore there is no need for legislation specifically dealing with GM foods. In this case, regulation focuses on the nature of the final food product rather than the process by which the food product is made [129]. Thus, products of GM crops and products of crossbreeding undergo the same regulatory processes.

While it must be stressed that some of the concerns raised about GM crops are founded and should be addressed prior to release, a lot of the opposition is either out of ignorance or it is outright manipulative. For example, as discussed in this review, there are claims that GM crops adversely affect humans and animals [81], yet there is no empirical data supporting this assertion. Furthermore, suggestions that transgenes will contaminate the environment appear to assume that transgenes, including those derived from virus genomes, are by their nature harmful to the environment. Advances in deep sequencing have revealed widespread endogenization of viral genomes by plants [71,72,73], thus GE for virus resistance is not as unnatural as claimed.

It must be emphasized that much of the hostility towards GM crops has been from Europe, and that this attitude is usually relayed to developing countries, especially sub-Saharan Africa. To leverage new discoveries in genomics and biotechnology and improve crop production, the research community will need to properly communicate new technologies to the public. Incidentally, attitudes towards GM crops in Europe appear to be changing. For example, a recent survey of EU citizens by Eurobarometers shows an increasing acceptance of GM crops [132] and this should ease the way and support a positive change in the regulation and adoption of these crops for large-scale cultivation in the EU.

## Figures and Tables

**Table 1:** Virus resistant GM crops approved for commercialization in various countries.

Country	Year of Approval	Crop /Cultivated Area	Technology Company/Organization	Trade Name	Target Viruses	Gene Targeted	References
USA	1996	Squash *	Seminis & Monsanto	CZW3, ZW20	Cucumber mosaic virus (CMV), Watermelon mosaic virus (WMV), Zucchini yellow mosaic virus (ZYMV)	Coat protein	[16]
	1997	Papaya *	Cornell University and the University of Hawaii	55-1 (Rainbow & SunUp), 63-1	Papaya ringspot virus (PRSV)	Coat protein	[91,92]
	1998	Potato *	Monsanto	NewLeaf™Plus Russet Burbank	Potato leaf roll virus (PLRV)	Replicase and helicase	[93,94]
				Shepody NewLeaf™Y potato	Potato virus Y (PVY)	Coat protein	[94,95]
	2007	Plum	USDA-ARS	C-5	Plum pox virus (PPV)	Coat protein	[96,97]
	2009	Papaya *	University of Florida	X17-2	Papaya ringspot virus (PRSV)	Coat protein	[98]
China	1998	Sweet pepper	Beijing University	PK-SP01	Cucumber mosaic virus (CMV)	Coat protein	[99]
	1999	Tomato *	Beijing University	PK-TM8805R(8805R)	Cucumber mosaic virus (CMV)	Coat protein	[100]
	2006	Papaya *	Cornell University and the University of Hawaii	55-1(Rainbow & SunUp), 63-1	Papaya ringspot virus (PRSV)	Coat protein	[91,92]
Canada	1999	Potato *	Monsanto	Shepody NewLeaf™Y potato	Potato virus Y (PVY)	Coat protein	[94,95]
Brazil	2011	Bean *	EMBRAPA	BRSFc401 RMD	Bean golden mosaic virus (BGMV)	RNA of viral replication protein	[101,102]
Japan	2011	Papaya *	Cornell University and the University of Hawaii	55-1 (Rainbow & SunUp), 63-1	Papaya ringspot virus (PRSV)	Coat protein	[91,92]
Kenya	2021	Cassava	Danforth Plant Science Center, St. Louis, USA	Cassava Event-4046	Cassava brown streak virus (CBSV)	RNAi against CP	[51]

* GE crops available to growers for cultivation.

## Data Availability

Not applicable.
